# Rational design of bioinspired tissue adhesives

**DOI:** 10.1002/ctm2.784

**Published:** 2022-04-07

**Authors:** Navajit S Baban, Yong‐Ak Song

**Affiliations:** ^1^ Division of Engineering New York University Abu Dhabi Abu Dhabi United Arab Emirates; ^2^ Department of Chemical and Biomolecular Engineering Tandon School of Engineering New York University New York USA; ^3^ Department of Biomedical Engineering Tandon School of Engineering New York University New York USA

1

There are numerous clinical needs for adhesives, such as wound closure, tissue repair, haemostasis assistance, and implant fixation.^1^ However, attaining optimum adhesion to soft tissues while minimizing tissue damage presents a considerable clinical challenge, especially when tissues are wet.^2^ Although several tissue adhesives are currently in clinical use,^1^ these adhesives in practice are far from ideal. They have low hydrophilicity, limited biocompatibility, and antimicrobial properties with poor contact compliance, and they do not work with important soft connective tissue types like tendons and ligaments.

There are several types of these adhesives, each with different weaknesses. Synthetic pressure‐sensitive adhesives have poor properties in part because they primarily rely on viscoelasticity and surface chemistry. Viscous adhesives prevent clean separation,^3^ which transfers remnants to the attached surface, thus making the site infected. Further, they are prone to fouling by particulate contamination and are not reusable.^3^ Chemical‐based adhesives, such as cyanoacrylate, involve tissue‐specific reactive chemistry (exothermic covalent cross‐linking reaction) with undetermined curing time. Additionally, chemical adhesives can induce an intense inflammatory response due to the release of toxic chemicals like formaldehyde.^1^, ^2^ Clinically used fibrin sealants and hydrogels can effectively glue to wet tissue without causing a significant inflammatory response. However, they exhibit low adhesion strength due to poor cohesive properties and do not adhere firmly to tissues such as skin.^1^, ^2^ Biocompatible hydrogels can achieve enhanced adhesion through a chemical bond with specific tissues. However, chemical bonding can easily be fouled in the presence of blood, thus compromising the efficacy of chemical‐based adhesives in many surgical settings.^2^ Although universally used for mechanical fixation, sutures and staples pose considerable limitations like extended operating time, inaccessibility to small spaces, increased risk of wound infection, seromas following operations, scarring, and damage to nerves and blood vessels.^2^

Nature, as it often does, inspires better technological solutions. Natural adhesives like feet of different arthropods and vertebrates demonstrate elegant and sophisticated attachment ability to almost any surface.^4^ These adhesives not only show high adhesion capabilities but can also be detached quickly (tunable adhesion) and reused repeatedly.^3^ For example, lizards and tree frogs have all garnered the interest of scientists for their extraordinary ability to walk on a variety of surfaces in their habitat.^4^, ^5^ Their feet show sturdy yet tunable and reversible adhesion, which is acquired from hierarchical structures that split into finer and finer micropillars, which terminate as nanoscale spatula‐ or mushroom‐shaped caps.^3^ The related biomimetic studies revealed the physics behind the micropillar‐based adhesion phenomenon,^6^ which has provided new bioinspired ideas to improve tissue adhesives.

Further, natural interfaces display microscale sutured or form‐based microinterlocked geometries, which provide flexible and robust connectivity to ensure multiple essential functionalities.^7^ Inspired by natural microinterlocks, scientists have developed several biomimetic structures to gain desirable functionalities concerning bioinspired tissue adhesives, such as transdermal drug delivery, conformal organ machine interface, and stretchable electronic skins.^8^

What all the above bioinspired examples have in common is that they showcase mechanics‐mediated adhesion rather than surface reactive chemistry‐mediated adhesion. This feature can potentially provide design solutions to universal soft tissue adhesion problems, with minimal traumatic removal and damage, less risk of infection, and drug delivery. However, a precise understanding of bioadhesives’ inherent toughening mechanisms is a prerequisite to achieving this lofty goal.

Our recent study^9^ on lizard tail autotomy proposed such a physics‐based approach to shed light on one of the fascinating adhesion problems. Tail autotomy is a life‐saving escape strategy that lizards have evolved, whereby a lizard self‐amputates its tail to evade the predators/attackers. It is a prominent natural example of tunable adhesion where a tail remains sturdily connected to its body part but only quickly breaks when needed. The ease with which lizards perform autotomy depends on the anatomy of the muscle joint that connects the tail to their body. For three lizard species (*Hemidactylus flaviviridis, Cyrtopodion scabrum* and *Acanthodactylus schmidti*), we found the muscle fracture planes (from the tail part) contained mushroom‐shaped microscopic pillars, whose tops included nanoscopic pores, as shown schematically in Figure 1. Proteomic studies and scanning electron microscopy of the fractured planes showed that the microscopic micropillars formed surface contact‐based attachment through adhesion forces rather than form‐based mechanical interlocking with the counter body part that might have made tail shedding more strenuous.

**FIGURE 1:**
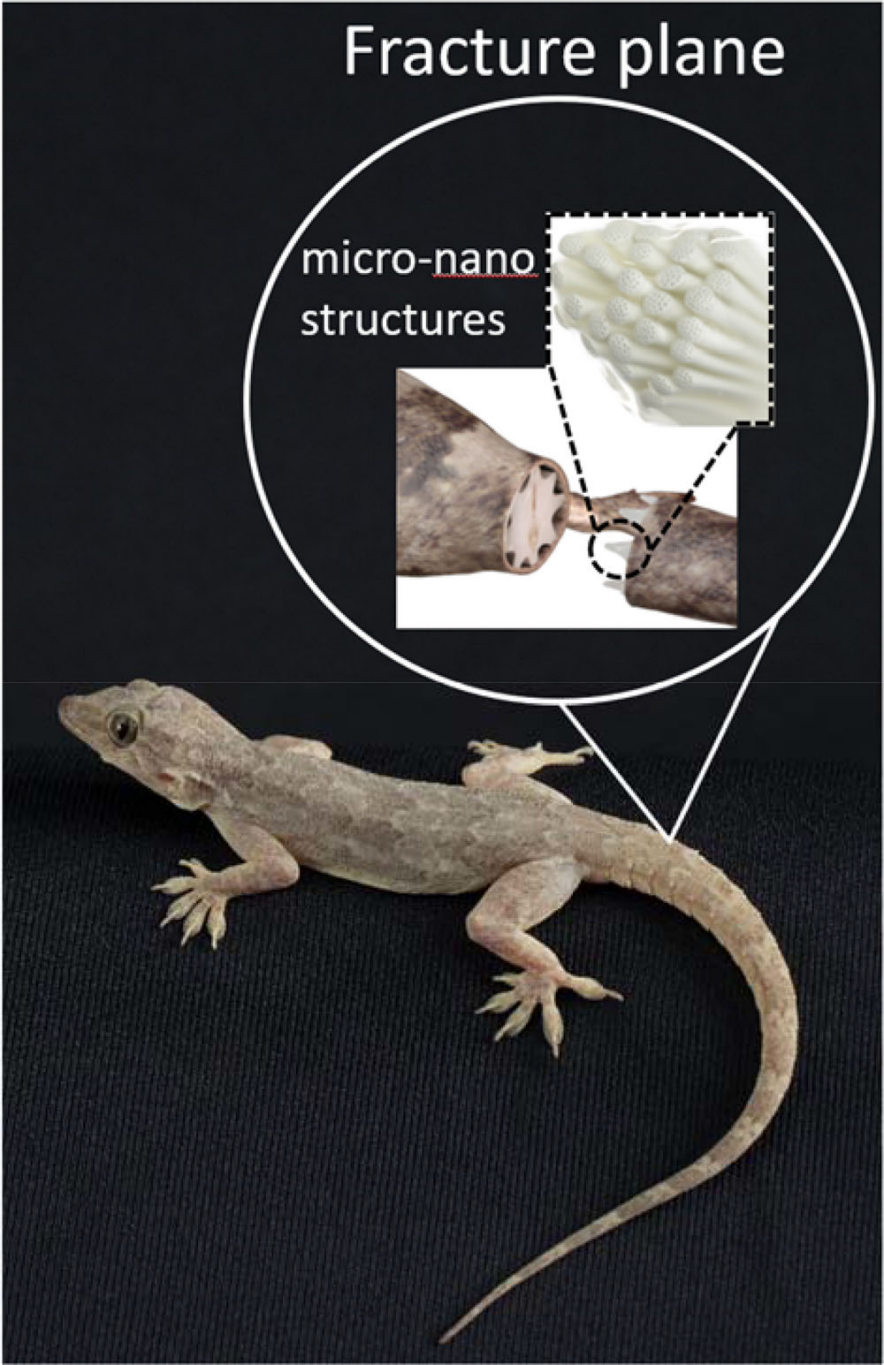
Schematic showing plug and socket connections between the tail and body facilitated by micro–nano structures along the fracture plane of *Hemidactylus flaviviridis* lizard

Using high‐speed videography, we found that these adhesive forces were strong enough to keep the tail intact during pulling. However, when the lizard bent its tail, it initiated a crack that swiftly propagated, leading to a quick separation of the sieged tail portion. Experiments with the biomimetic model in dry conditions showed that the micropillars with nanopores enhanced adhesion 15‐fold more than the planar control. In wet conditions, liquid micro‐ and nanobridges between the contacting surfaces increased adhesion further through elastocapillarity effects similar to beetle *Hemisphaerota cyanea*.^4^ Related physics‐based computer models revealed underlying micro‐ and nanoscale toughening mechanisms^9^: Cook‐Gordon mechanism (C‐G, repulsive interaction between cracks), Lake–Thomas effect (L‐T, intermittent crack propagation with energy dissipation), and micropillar's height‐based strain energy absorption. The combined action of all the above‐mentioned toughening mechanisms enhanced adhesion of the micropillars with nanoporous tops to the body part and counterbalanced facile loss of the tail. However, when switching from pulling to bending, the whole fracture process eased 17‐fold in the bent mode compared to the tensile mode because the load shared by the micropillars decreased accordingly, facilitating quick tail release. Further, our computational simulation showed a significant influence of muscle contraction, which tends to relax the compressive stress around the connecting surface for easy release. In a follow‐up study,^10^ we biomimicked lizard tail autotomy and related muscle contraction using soft microinterlocking structures^7^ that can be actuated on‐demand via subsurface soft patch, showing high‐end tunable adhesion. Here too, the computer modelling identified C‐G and L‐T as the inherent toughening mechanisms. Such micro‐ and nanostructure‐based tunable adhesion patches could potentially be applied as tissue adhesives in clinical applications where controlled tunability is required.

Most importantly, our study suggested that understanding and quantifying the basic physical toughening mechanisms such as C‐G and L‐T through modelling and computational simulation of the bioinspired adhesive models can provide a more rational design approach in the quest to overcome existing adhesives’ limitations. This approach can help bioengineers and scientists to design and optimize the bioinspired tissue adhesives in terms of their geometrical parameters for various biomedical applications in a more time‐ and cost‐effective manner.

## CONFLICT OF INTEREST

The authors declare no conflict of interest.
